# Outreach and Inreach Organized Service Screening Programs for Colorectal Cancer

**DOI:** 10.1371/journal.pone.0155276

**Published:** 2016-05-12

**Authors:** Chu-Kuang Chou, Sam Li-Sheng Chen, Amy Ming-Fang Yen, Sherry Yueh-Hsia Chiu, Jean Ching-Yuan Fann, Han-Mo Chiu, Shu-Lin Chuang, Tsung-Hsien Chiang, Ming-Shiang Wu, Chien-Yuan Wu, Shu-Li Chia, Yi-Chia Lee, Shu-Ti Chiou, Hsiu-Hsi Chen

**Affiliations:** 1 Department of Internal Medicine, College of Medicine, National Taiwan University, Taipei, Taiwan; 2 Division of Gastroenterology and Hepatology, Chia-Yi Christian Hospital, Chia-Yi, Taiwan; 3 School of Oral Hygiene, College of Oral Medicine, Taipei Medical University, Taipei, Taiwan; 4 Department and Graduate Institute of Health Care Management, Chang Gung University, Tao-Yuan, Taiwan; 5 Department of Health Industry Management, Kainan University, Tao-Yuan, Taiwan; 6 Institute of Epidemiology and Preventive Medicine, College of Public Health, National Taiwan University, Taipei, Taiwan; 7 Department of Integrated Diagnostics and Therapeutics, National Taiwan University Hospital, Taipei, Taiwan; 8 Graduate Institute of Clinical Medicine, College of Medicine, National Taiwan University, Taipei, Taiwan; 9 Health Promotion Administration, Ministry of Health and Welfare, Taipei, Taiwan; 10 Institute of Public Health, National Yang-Ming University, Taipei, Taiwan; University of Arizona, UNITED STATES

## Abstract

**Background:**

Outreach (*i*.*e*., to invite those who do not use, or who under use screening services) and inreach (*i*.*e*., to invite an existing population who have already accessed the medical system) approaches may influence people to increase their use of screening test; however, whether their outcomes would be equivalent remains unclear.

**Methods:**

A total of 3,363,896 subjects, 50–69 years of age, participated in a colorectal cancer (CRC) screening program using biennial fecal immunochemical tests; 34.5% participated during 2004–2009 when the outreach approach alone was used, and 65.5% participated from 2010–2013 when outreach was integrated with an inreach approach. We compared the outcomes of the two approaches in delivery of screening services.

**Results:**

Coverage rates increased from 21.4% to 36.9% and the positivity rate increased from 4.0% to 7.9%, while referral for confirmatory diagnostic examinations declined from 80.0% to 53.3%. The first period detected CRC in 0.20% of subjects screened, with a positive predictive value (PPV) of 6.1%, and the second detected CRC in 0.34% of subjects, with a PPV of 8.0%. After adjusting for confounders, differences were observed in the PPV for CRC (adjusted relative risk, 1.50; 95% confidence interval [CI], 1.41–1.60), cancer detection rate (1.20; 95% CI, 1.13–1.27), and interval cancer rate (0.72; 95% CI, 0.65–0.80). When we focused on the comparison between two approaches during the same study period of 2010–2013, the positivity rate of fecal testing (8.2% vs. 7.6%) and the PPV for CRC detection remained higher (1.07; 95% CI, 1.01–1.12) in subjects who were recruited from the inreach approach.

**Conclusions:**

Outcomes of screening were equivalent or better after integration of outreach and inreach approaches.

**Impact:**

The results will encourage makers of health-care policy to adopt the integration approach to deliver screening services.

## Introduction

Colorectal cancer (CRC) poses a significant threat to global health [1]. Because mass screening based on either fecal occult-blood test [2–5], sigmoidoscopy [6, 7], or colonoscopy [8–11] has the potential to reduce mortality from CRC, mass screening has been adopted as a national policy in many countries. Fecal occult-blood testing, especially fecal immunochemical testing (FIT), has increasingly gained popularity in the eligible screenees whose adherence to primary colonoscopy is poor and/or where the availability of colonoscopist is scare [12–15].

The use of FIT is not without drawbacks. A FIT screening program is based on a two-stage design. The first stage is to reach the population and to collect and analyze stool samples, and the second stage is the administration of colonoscopy to those who test positive; both stages require monitoring of screening indicators to ensure quality [16]. Both outreach and inreach approaches are used to increase the uptake of FITs in the population, which is regarded as an important health promotion behavior for enhancing the compliance. Outreach seeks subjects in the community who do not use or who underuse medical services through the use of mail, telephone calls, mass media, and a CRC awareness campaign, and delivers on-site screening service as the incentive to encourage them to participate in screening [17] whereas inreach provides preventive services to those who have already engaged in the health-care system for treatment unrelated to screening for CRC [18, 19], in which personalized assessment is possible through the face-to-face discussion of his/her personal health history and clinical symptom with the physician to determine whether the individual should undergo CRC screening [20, 21]. In Europe, most of the national programs adopt an outreach approach by inviting eligible subjects through mailing invitation letters with or without stool tests [22–27]. While in the US, for example the Colorectal Cancer Control Program, screening services are not only promoted to beneficiaries of insurance using both outreach and inreach approaches [28, 29], but are also provided via an outreach approach to those persons who encounter barriers to accessing the health-care system or who live in areas with a high proportion of uninsured persons [30].

There are several differences between these two approaches. For example, the outreach approach may enroll a higher proportion of asymptomatic subjects and when cancer is detected, it tends to be of an earlier stage; by contrast, in the inreach approach, a higher prevalence of clinical symptoms may be associated with a greater likelihood of advanced cancers, which may be beyond the stage of early detection. In addition, in subjects who test positive, those ascertained through the outreach approach are more likely to encounter obstacles in the referral process than are those ascertained through the inreach approach. Inreach tends to avoid fragmentation of professional responsibility, and thus, a confirmatory examination may be arranged in a timely manner [31].

To accommodate the needs of subpopulations, different approaches to distribute the screening services may be chosen, especially when an organized screening is conducted on a nationwide scale. Although there is general agreement that everyone should have equal access to the same standard of screening, and that everyone should receive the same benefits from early detection, there is no solid evidence that inreach and outreach approaches would achieve the same outcomes, given their fundamental differences in population characteristics and screening process. To evaluate the outcomes of mass screening, a series of indicators are required. They may include the positive predictive value (PPV) for cancer detection, cancer detection rate, interval cancer rate, and CRC staging by detection modes; however, without a large population-based longitudinal follow-up cohort, a thorough evaluation employing all of these measures is difficult.

In Taiwan, similar to many countries in the Asia-Pacific region, there has been a substantial increase in the burden of CRC. Starting 2004, a nationwide CRC screening program has been launched using the outreach approach [3, 4]. In 2010, aiming to improve the accessibility of screening, an inreach approach was further added within the already established outreach system. With this unique dataset based on a nationwide cohort, the present study tested the hypothesis that the performance of mass screening would be equivalent before and after the integration of two different approaches by a thorough evaluation of the outcome indicators.

## Methods

### Taiwanese Nationwide CRC Screening Program

This nationwide program was started in 2004 by inviting subjects aged 50–69 years to undergo biennial FIT, which was funded by the Health Promotion Administration, Ministry of Health and Welfare (formerly Bureau of Health Promotion). Screening included a stepwise protocol, including the invitation of potential participants, distribution of FIT kits, storage, transportation, and analysis of FIT, referral for colonoscopy for those who tested positive, and histopathological diagnosis. All results were transmitted to a central database via a virtual private network such that the standardized indicators could be generated periodically to monitor the performance of screening [3, 4].

### Delivery methods of screening service

The nationwide program can be divided into two periods differing by approach to the delivery of screening tests. The first period (Period 1), from 2004 to 2009, used the outreach approach only, and the second period (Period 2), from 2010 to the present time, retained the outreach approach and integrated an inreach approach into the existing framework. These approaches are described as follows:

#### The outreach approach

The nationwide program started with reaching out to eligible subjects in 25 municipalities in Taiwan [4].The screening service was audited by the local Public Health Bureau in each municipality, where the FITs were distributed to eligible individuals by Public Health Units scattered in the municipal districts. During the study period, approximately 333 units nationwide identified eligible persons by using the population registry. Individuals were encouraged to participate in the screening through the use of mail, telephone calls, mass media, and a CRC awareness campaign. After participants completed the fecal sampling, the samples were returned to Public Health Units for analysis; subjects with positive screening test results were referred to the hospitals for confirmatory diagnostic examinations and their outcomes were be tracked and recorded.

#### The inreach approach

Starting 2010, an inreach approach was added to the established outreach system by inviting those who were using medical services to participate in screening. The Health Promotion Administration included qualified hospitals and clinics in each municipality as screening units in the nationwide program. The number of participating hospitals and clinics increased from 1,501 in 2010 to 3,277 in 2013. With this approach, information related to the CRC screening was displayed via posters or video tapes in hospital or clinic waiting rooms, reminding both patients and persons accompanying patients to undergo CRC screening, and encouraging them to discuss CRC screening with their primary care physician in a personalized manner. During consultation, physicians and nurses would prompt eligible subjects to participate in screening and also, the patients could be self-motivated to request the screening test. When their FITs showed positive results, confirmatory diagnostic procedures would be arranged by the physicians.

### FIT

The biennial one-day method was adopted, and the choice of FIT kit was based on a local bidding process by each Public Health Bureau or hospital/clinic. Two major brands were available, including the OC-Sensor (Eiken Chemical Co, Tokyo, Japan) and the HM-Jack (Kyowa Medex Co Ltd, Tokyo, Japan) tests; both were quantitative and their cutoff values for a positive test were 100 and 12 ng hemoglobin/mL buffer (8 ng/mL during the period of 2004–2009 for HM-JACK), respectively. Test for equivalence between two tests has been reported previously [3]. Subjects were asked to submit the stool samples immediately after they were obtained. The analyses of FIT were performed at approximately 125 laboratories certified according to ISO 15189 quality standards.

### Confirmatory examination for positive FIT and follow-up

Subjects with positive FIT were referred to the hospitals for confirmatory examination with either colonoscopy or sigmoidoscopy plus barium enema (only in cases when the colonoscopy was not feasible or was declined by the subjects); it was recommended that confirmatory diagnostic examinations be performed within 3 months. The diagnostic details, including the size, location, and histopathology for colonic neoplasms were recorded. The histopathology was classified according to the criteria of the World Health Organization [32].

Standardized process indicators were evaluated periodically. These included the coverage rate (number of screened subjects/total number of subjects eligible for screening), the positivity rate (number of positive FITs/total number of FITs), referral rate of diagnostic examinations (number of colonoscopies or other diagnostic examinations performed/total number of positive FITs), and time to confirmatory examinations.

### Evaluation of outcome

First, we made a comparison between the Period 1 and Period 2 over the various process and outcome indicators. Second, because the increasing incidence of CRC and possibly more aggressive screening efforts or other structural changes in the health system may happen during the long study period, which could confound the comparison of outcome indicators between these two periods, we separated the results of Period 2 according to the different screening unit that provided the outreach or inreach approach and repeated the analyses in order to control this so-called historical effect (S1 File).

#### PPV and detection rate

Outcome indicators were evaluated based on data from the prevalent screen. The first indicators included the PPV of cancer (number of subjects with CRC/total number of diagnostic endoscopies) and the cancer detection rate (number of subjects with CRC/number of subjects in the tested population). The detection of advanced adenoma, defined as an adenoma of ≥10 mm in diameter or having a villous component or high-grade dysplasia, was included in the calculations for the indicators as above [32].The per-person analysis was used for both the CRC (*i*.*e*., an individual discovered with metachronous cancers counted as one individual with cancer) and advanced adenoma (*i*.*e*., the most advanced finding being an advanced adenoma).

#### Interval cancer rate and test sensitivity

The second indicator was the interval cancer rate (*i*.*e*., the number of CRCs diagnosed after a negative FIT and <2 years to the next screen/total person-years at risk) [33].To ascertain the occurrence of interval cancer, the screening database was linked with the Taiwan Cancer Registry (2004–2013), a nationwide program with high coverage (99%; each hospital mandated to report all cases of CRC) and high accuracy (percentage of death-certificate-only cases of less than 1% for CRC) [34]. The indicator of test sensitivity was generated from the number of interval cancers using the proportional incidence method based on age- and gender-specific incidence derived from the Taiwan Cancer Registry [3].To consider adherence to the screening process, the two-year sensitivity of the screening program was also evaluated by including in the calculation of interval cancers those who had positive FIT findings followed by a negative assessment or no further assessment.

#### Cancer staging by detection modes

Third, the detection of CRC could be classified according to four different detection modes, including CRC detected by screening, subsequent screen-detected CRC, interval cancer, and CRC in non-participants. The distribution of cancer staging among these four groups could be treated as a surrogate for CRC-specific mortality. To ascertain the staging of incident CRCs in this cohort, we linked the screening database with Taiwan Cancer Registration, where the American Joint Committee on Cancer (AJCC) 7^th^ scheme was used for cancer staging [35].

### Statistical analysis

Differences in baseline characteristics and process indicators between the two periods were determined by applying the Student *t* or χ^2^ test. For the univariate analyses of outcome indicators, the two-sample proportion test was used to compare the PPV and detection rates of cancer and advanced adenoma. Because advanced age and male gender are well-recognized risk factors for colorectal neoplasms [36], results stratified according to these two factors are also reported. For the comparisons of interval cancer rate and test sensitivity, the Poisson regression method was used.

To adjust for differences in age, gender, brand of FIT, city/county, and the quality of colonoscopy between the two periods, we performed a multi-variable Poisson regression analysis with the outcome variables of PPVs for advanced adenoma detection and cancer detection, advanced adenoma and cancer detection rates, and interval cancer rate. The results are expressed as the adjusted relative risk (RR) and the corresponding 95% confidence interval (CI). We used the hospital level (*i*.*e*., medical center/regional hospital *vs*. local hospital/clinic) as a surrogate for quality of colonoscopy; the justification for this is described in **Table A in S1 File**.

To compare the distribution of cancer stage (at detection) by the detection modes, the Poisson method was used. We hypothesized that mass screening would efficiently detect CRC at the earlier stages such that there would besignificant changes in cancer stage distribution between the screening participants and non-participants.

All statistical analyses were performed using SAS version 9.4 (SAS Institute, Cary, NC, USA). All *P* values were 2-sided, and a *P* value <0.05 was considered to be statistically significant.

### Ethics

This study was approved by the Health Promotion Administration, Ministry of Health and Welfare prior to data retrieval and analysis (1049903864) and Research Ethics Committee of National Taiwan University Hospital (201511034W). Patient records/information was anonymized and de-identified prior to analysis.

## Results

### Baseline characteristics

From January 1, 2004 to December 31, 2013, a total of 3,363,896 subjects took part in the nationwide program, consisting of 1,160,895 and 2,203,001 subjects in the Period 1 and Period 2, respectively. The coverage rate increased from 21.4% in the period 1 to 36.9% % in the period 2 given the corresponding eligible populations of 5,417,699 and 5,976,667, respectively (**Table 1**). Differences were also salient in the percentage of male participants, the level of hospital where confirmatory diagnostic testing was performed, confirmatory examination tools, number of screen-detected cancers, and the colonoscopic quality indicators (including cecal intubation rate, adenoma detection rate, advanced adenoma detection rate, and resection rate of adenoma <2cm; see **Table A in S1 File**). Small differences, albeit statistically significant owing to the large sample size, were observed with respect to geographic areas and the brands of FIT used. There was lacking of significant difference in the mean age or time to confirmatory diagnostic examination.

**Table 1:** Baseline characteristics of the screened population.

Characteristics	Period 1 (*n* = 1,160,895)	Period 2 (*n* = 2,203,001)	*P* value
**Demographic characteristics**			
Coverage rate (%)	21.4	36.9	<0.01
Age, years (mean ± SD)	58.62 ± 5.84	57.55 ± 5.63	0.89
Gender, *n* (%)			<0.01
Male	446,290 (38.4)	1,023,649 (46.5)	
Female	714,605 (61.6)	1,179,352 (53.5)	
**Geographic area, *n* (%)**			<0.01
Northern area	460,668 (39.7)	938,757 (42.6)	
Central area	289,309 (24.9)	514,840 (23.4)	
Southern area	339,322 (29.2)	634,022 (28.8)	
Eastern area and offshore island	71,596 (6.2)	115,382 (5.2)	
**Fecal immunochemical test, *n* (%)**			<0.01
OC-Sensor	747,076 (64.4)	1,382,364 (62.7)	
HM-Jack	208,929 (18.0)	820,627 (37.3)	
Others	204,890 (17.6)	10 (0.0)	
**Confirmatory examination characteristics**			
Time to confirmatory examination, months (mean ± SD)	1.17 ± 1.47	1.31 ± 1.54	0.95
Hospital level for confirmatory diagnosis, *n* (%)			<0.01
Medical center	8,786 (23.4)	33,613 (36.1)	
Regional hospital	16,863 (44.9)	40,306 (43.3)	
Local hospital and clinic	9,545 (25.4)	17,811 (19.2)	
Non-specified	2,391 (6.3)	1,328 (1.4)	
Confirmatory examination tool, *n* (%)			<0.01
Colonoscopy	32,137 (85.5)	85,141 (91.5)	
Sigmoidoscopy± barium enema	5,116 (13.6)	7,699 (8.3)	
Missing data	332 (0.9)	218 (0.2)	
Screen-detected cancer, *n* (per 1,000)	2,304 (2.0)	7,479 (3.4)	<0.01
Colonoscopic quality indicator (%)			
Cecal intubation rate*	79.0	93.2	<0.01
Adenoma detection rate^†^	44.5	54.2	<0.01
Advanced adenoma detection rate^†^	13.8	17.2	<0.01
Resection rate of <2cm adenoma^‡^	84.9	91.1	<0.01

*Cecal intubation rate was defined as the number of subjects with cecal intubation / the number of subjects receiving colonoscopy

^†^(advanced) adenoma detection rate was defined as the number of subjects with at least one detected (advanced) adenoma/the number of subjects positive to FIT having attended a colonoscopy

^‡^resection rate of <2cm adenoma was defined as the number of subjects with resection of adenoma/the number of subjects with at least one detected <2cm adenoma having attended a colonoscopy.

When the analyses were restricted to Period 2 (**Table B in S1 File**), the coverage rates were 16.1% and 20.8%, respectively, for the groups recruited from outreach and inreach approaches. Regarding the evaluation of which sub-populations may have responded to the two different approaches, no significant difference was noted in the mean age (57.4 *vs*. 57.6 years) and the difference in the percentage of male participants was small (46% *vs*. 46.9%).

The difference in confirmatory examination tools, number of screen-detected cancers, and the colonoscopic quality indicators were also decreased while the magnitude of difference remained similar in the geographic area and hospital level for confirmatory diagnosis, which might be related to the relative constancy in the geographic distribution of hospital facility supplies during the study period.

### Positivity rate and referral rate

The positivity rate increased from Period 1 (4.0%) to Period 2 (7.9%) (**Table 2**); however, for subjects who tested positive, the referral rate for confirmatory diagnostic examinations declined from 80.0% to 53.3%. With regard to age- and gender-specific rates, as expected, the positivity rates of FIT were higher in men and in older age groups, observed similarly in both periods. The referral rates were similar across different strata according to age and gender.

**Table 2:** Numbers of tested population, positive tests, and confirmatory diagnoses stratified by the age, gender, and the periods of the nationwide colorectal cancer screening program.

	Tested population		Positive test		Positivity rate (%)		Diagnostic examination		Referral rate for diagnostic examination (%)	
Period	Period 1	Period 2	Period 1	Period 2	Period 1	Period 2	Period 1	Period 2	Period 1	Period 2
Male										
50–59 years	258,147	663,275	10,849	57,976	4.2^†^	8.7^†^	8,711	31,353	80.3^†^	54.1^†^
60–69 years	188,143	360,374	11,570	43,391	6.1^†^	12.0^†^	9,247	23,190	79.9^†^	53.4^†^
Subtotal	446,290	1,023,649	22,419	101,367	5.0^†^	9.9^†^	17,958	54,543	80.1^†^	53.8^†^
Female										
50–59 years	458,523	831,295	13,800	45,207	3.0^†^	5.4^†^	11,161	24,297	80.9^†^	53.7^†^
60–69 years	256,082	348,057	10,744	28,056	4.2^†^	8.1^†^	8,466	14,218	78.8^†^	50.7^†^
Subtotal	714,605	1,179,352	24,544	73,263	3.4^†^	6.2^†^	19,627	38,515	80.0^†^	52.6^†^
Both genders										
50–59 years	716,670	1,494,570	24,649	103,183	3.4^†^	6.9^†^	19,872	55,650	80.6^†^	53.9^†^
60–69 years	444,225	708,431	22,314	71,447	5.0^†^	10.1^†^	17,713	37,408	79.4^†^	52.4^†^
Total	1,160,895	2,203,001	46,963	174,630	4.0^†^	7.9^†^	37,585	93,058	80.0^†^	53.3^†^

*P <0.05 or

^†^*P* <0.01 in the comparison between two periods.

The comparison between outreach and inreach groups in Period 2 (**Table C in S1 File**) showed that the FIT positivity rate remained higher in the inreach group (8.2% *vs*. 7.6%). A lower referral rate for diagnostic examination was similarly seen in both groups.

### PPV and detection rate

In Period 1, CRC was detected in 0.20% of patients, with a PPV of 6.1%; in Period 2 CRC was detected in 0.34% of patients, with a PPV of 8.0%.The PPVs and the cancer detection rates were significantly higher in Period 2 than Period 1 (**Table 3**). PPVs and cancer detection rates were also higher for male gender and older age groups as compared with the total population group. When advanced adenoma was used as the index lesion, the findings were similar: PPVs and detection rates for advanced adenoma were also greater in Period 2, in men, and in older age groups.

**Table 3:** Positive predictive values and detection rates for the advanced adenoma and colorectal cancer according to the age, gender, and periods of the nationwide colorectal cancer screening program.

	Positive predictive value (%)				Detection rate(per 1,000)			
	Advanced adenoma		Colorectal cancer		Advanced adenoma		Colorectal cancer	
Period	Period 1	Period 2	Period 1	Period 2	Period 1	Period 2	Period 1	Period 2
Male								
50–59 years	15.8^†^	18.5^†^	5.5^†^	6.9^†^	5.3^†^	8.7^†^	1.8^†^	3.3^†^
60–69 years	16.2^†^	20.1^†^	8.5^†^	10.9^†^	8.0^†^	13.0^†^	4.2^†^	7.0^†^
Subtotal	16.0^†^	19.2^†^	7.0^†^	8.6^†^	6.4^†^	10.2^†^	2.8^†^	4.6^†^
Female								
50–59 years	7.7^†^	10.4^†^	4.7^†^	6.4^†^	1.9^†^	3.0^†^	1.1^†^	1.9^†^
60–69 years	9.1^†^	12.9^†^	6.1^†^	8.7^†^	3.0^†^	5.3^†^	2.0^†^	3.6^†^
Subtotal	8.3^†^	11.3^†^	5.3^†^	7.2^†^	2.3^†^	3.7^†^	1.5^†^	2.4^†^
Both genders								
50–59 years	11.3^†^	15.0^†^	5.0^†^	6.7^†^	3.1^†^	5.6^†^	1.4^†^	2.5^†^
60–69 years	12.8^†^	17.4^†^	7.4^†^	10.1^†^	5.1^†^	9.2^†^	2.9^†^	5.3^†^
Total	12.0^†^	15.9^†^	6.1^†^	8.0^†^	3.9^†^	6.7^†^	2.0^†^	3.4^†^

*P <0.05 or

^†^*P* <0.01 in the comparison between two periods.

When we focused on the comparison between two groups in Period 2, the PPVs (8.3% *vs*. 7.7%) and cancer detection rates (3.5 *vs*. 3.3 per 1,000) were still higher in the inreach group as compared with the outreach group (**Table D in S1 File**).

### Interval cancer rate and test sensitivity

As shown in **Table 4**, the interval cancer rate for Period 2 was lower than that for Period 1 (23.5 *vs*. 33.4per 100,000 person-years), resulting in a significant difference in test sensitivities (75% *vs*. 66%, *P* <0.01). The test sensitivity for each period was, however, similar among different subgroups according to gender and age. To consider adherence to the recommendation of confirmatory diagnostic testing, the two-year sensitivity of the screening program was evaluated by including in the calculation of interval cancers those individuals who had positive FIT findings followed by a negative assessment or no further assessment. Using this approach, no significant difference was observed between the two periods (Period 2: 61% *vs*. Period 1: 62%).

**Table 4:** Comparisons of the number of interval cancer, interval cancer rate, and test sensitivity between two periods of the nationwide colorectal cancer screening program.

	Person-year at risk*	No. of IC	Incidence of IC(expected incidence in the absence of screening)^†^	Proportional incidence	Test sensitivity^1^, %(95% CI)^‡^	Two-year sensitivity^2^, % (95% CI)^‡^
Period 1						
Male						
50–59 years	498,915	134	26.9 (72.9)	0.37	63 (57–70)	57 (52–64)
60–69 years	383,418	240	62.6 (177.5)	0.35	65 (60–70)^§^	60 (56–65)
Subtotal	882,333	374	42.4 (118.3)	0.36	64 (60–68)^§^	59 (56–63)
Female						
50–59 years	927,478	176	19.0 (53.1)	0.36	64 (59–70)	60 (55–66)
60–69 years	539,543	234	43.4 (129.4)	0.34	66 (62–72)^§^	63 (58–68)
Subtotal	1,467,021	410	27.9 (81.2)	0.34	66 (62–69)^§^	62 (58–65)
Both genders						
50–59 years	1,426,393	310	21.7 (62.9)	0.35	65 (61–70)	61 (57–65)
60–69 years	922,961	474	51.4 (152.6)	0.34	66 (63–70)^§^	62 (59–65)
Total	2,349,354	784	33.4 (98.1)	0.34	66 (63–69)^§^	62 (59–64)
Period 2						
Male						
50–59 years	1,152,145	253	22.0 (75.3)	0.29	71 (66–76)	54 (51–58)
60–69 years	651,882	273	41.9 (180.3)	0.23	77 (73–81)^§^	61 (58–65)
Subtotal	1,804,027	526	29.2 (113.2)	0.26	74 (71–78)^§^	58 (56–61)
Female						
50–59 years	1,501,811	225	15.0 (55.0)	0.27	73 (68–78)	61 (57–65)
60–69 years	645,073	179	27.7 (131.6)	0.21	79 (74–84)^§^	68 (64–73)
Subtotal	2,146,884	404	18.8 (78.0)	0.24	76 (72–80)^§^	64 (61–68)
Both genders						
50–59 years	2,653,956	478	18.0 (65.0)	0.28	72 (69–76)	58 (55–61)
60–69 years	1,296,955	452	34.9 (155.1)	0.22	78 (74–81)^§^	64 (61–67)
Total	3,950,911	930	23.5 (94.6)	0.25	75 (73–78)^§^	61 (59–63)

IC = interval cancer.

*The interval cancer was defined as a cancer that developed in the interval of 2 years following a negative FIT result. For those who had more than 2 years of follow-up but did not receive the subsequent screening, their follow-up time was set at 2 years in the calculation of person-years at risk.

^†^Per 100,000 person-years

^‡^Test sensitivity^1^was generated from the number of interval cancer in the two-year period of observation following a negative FIT. Two-year sensitivity^2^ of the program was generated from the number of interval cancer in the two-year period of observation following a negative FIT or a positive FIT followed by a negative assessment or no further assessment.

^§^*P* <0.01 in the comparison between outreach and inreach periods.

When we made a comparison between two groups in Period 2, however, significant difference was no longer seen in the interval cancer rate or test sensitivity (**Table E in S1 File**), which may indicate that the difference in the comparison between Period 1 and Period 2 was related to improvement of the screening test over time, rather than the use of outreach or inreach approach.

### Multivariate analysis

Taking into account the differences in baseline characteristics of the two screening periods, multivariate analyses with adjustment for demographics, geographic areas, and hospital level (a surrogate for the quality of confirmatory diagnostic examinations) were performed. The results are shown in **Table 5,** and the findings were remarkably similar to those obtained from the univariate analyses: for either CRC or advanced adenoma, a higher PPV, a higher detection rate, and a lower interval cancer rate were noted in Period 2 as compared with Period 1.

**Table 5:** Comparisons of the test performance between two periods of the nationwide colorectal cancer screening program using the Poisson regression models.

Model*	Relative risk	95% CI
Positive predictive value for advanced adenoma detection		
Model 1		
Period 2 *vs*. period 1	1.33	1.28–1.37^†^
Model 2		
Period 2 *vs*. period 1	1.21	1.14–1.28^†^
Age 60–69 *vs*. 50–59 years	1.12	1.09–1.16^†^
Male *vs*. female	1.71	1.65–1.76^†^
OC-Sensor *vs*. HM-Jack	1.19	1.14–1.25^†^
Medical center/regional hospital *vs*. local hospital/clinic^‡^	0.95	0.88–1.02
Advanced adenoma detection rate		
Model 1		
Period 2 *vs*. period 1	1.73	1.68–1.79^†^
Model 2		
Period 2 *vs*. period 1	0.95	0.89–1.01
Age 60–69 *vs*. 50–59 years	1.15	1.48–1.57^†^
Male *vs*. female	1.83	2.56–2.75^†^
OC-Sensor *vs*. HM-Jack	1.09	0.93–1.96
Medical center/regional hospital *vs*. local hospital/clinic^‡^	1.31	1.19–1.43^†^
Positive predictive value for cancer detection		
Model 1		
Period 2 *vs*. period 1	1.32	1.26–1.38^†^
Model 2		
Period 2 *vs*. period 1	1.50	1.41–1.60^†^
Age 60–69 *vs*. 50–59 years	1.47	1.40–1.53^†^
Male *vs*. female	1.16	1.12–1.21^†^
OC-Sensor *vs*. HM-Jack	1.29	1.22–1.36^†^
Medical center/regional hospital *vs*. local hospital/clinic^‡^	1.09	1.01–1.18^†^
Cancer detection rate		
Model 1		
Period 2 *vs*. period 1	1.71	1.63–1.79^†^
Model 2		
Period 2 *vs*. period 1	1.20	1.13–1.27^†^
Age 60–69 *vs*. 50–59 years	1.50	1.43–1.56^†^
Male *vs*. female	1.24	1.20–1.29^†^
OC-Sensor *vs*. HM-Jack	1.18	1.12–1.24^†^
Medical center/regional hospital *vs*. local hospital/clinic^‡^	1.44	1.34–1.55^†^
Interval cancer rate		
Model 1		
Period 2 *vs*. period 1	0.71	0.64–0.78^†^
Model 2		
Period 2 *vs*. period 1	0.72^†^	0.65–0.80^†^
Age 60–69 *vs*. 50–59 years	2.06^†^	1.85–2.30^†^
Male *vs*. female	1.48^†^	1.33–1.64^†^
OC-Sensor *vs*. HM-Jack	0.91	0.81–1.02

*Model 1: the crude Poisson regression model; model 2: the multivariate Poisson regression model adjusted for the city/county clustering, age and gender distributions, brand of FIT, and the hospital levels^‡^ (a dichotomous predictor to represent the colonoscopy quality for positive predictive value and detection rate).

^†^*P*<0.05.

Regarding the comparison between outreach and inreach groups in Period 2 (**Table F in S1 File**), the PPV for CRC remained higher in the inreach group after adjustment of potential confounders.

### Cancer staging by detection mode

**Fig 1:**
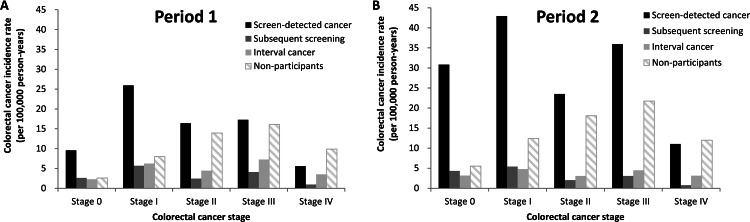
Incidence rates of CRC of the nationwide screening program based on biennial FIT, which are stratified by the study periods and the cancer detection modes. Cancer was staged according to the American Joint Committee on Cancer (AJCC) 7^th^ staging system.

Cancer incidence rates stratified by the cancer staging and detection modes are shown in **Fig 1**. In both periods, the distribution towards earlier in stages was seen among screen-detected cancers, subsequent screen-detected cancers, and interval cancers than that of the non-participants (*P* <0.01). When we made a comparison between two periods, we found that, among the screen-detected cancers, there were substantial increases in the incidence rates of stage 0 and 1 CRCs in Period 2; however, there were also significant increases in the stage 3 and 4 CRCs. By contrast, there were only modest increases in the incidence rates of all stages of CRC among the subsequent screen-detected cancers, interval cancers, and cancers in non-participants.

For the comparison between outreach and inreach groups in Period 2, the findings of early-stage cancer detection were similarly seen in two groups (**Fig A in S1 File**) while the numbers of cancer detection appeared to be higher in the inreach group.

## Discussion

In the present study, the performance of two different approaches in the delivery of screening for CRC was thoroughly evaluated using a large-scale population-based dataset, which generated several important findings. First, within an existing outreach system, the implementation of an inreach approach was proven to be effective in increasing the coverage rate of screening through the improvement in accessibility. Second, by including those who have utilized the health-care system, the inreach approach has identified a higher proportion of individuals with positive FIT results and among them, colorectal neoplasms were indeed prevalent. Third, when we took into consideration the historical effect by comparing two approaches in the same study period, the higher FIT positivity rate and PPV for cancer detection were similarly seen in the inreach group, which indicated that through the face-to-face assessment, subjects with higher risk of CRC were more likely to be identified and invited to participate in the mass screening. Fourth, in both periods, the majority of screen-detected CRCs were early in stage and following curative treatment, a substantial reduction of CRC-specific mortality is anticipated.

Methods to increase the coverage of screening may be categorized into (1) how to improve access to screening and (2) how to include the difficult-to-reach population. Regarding the former, as shown in **Table G in S1 File**, the reported coverage rates fluctuated, ranging from 17% to 68% [23–26, 29, 37–41]. Instead of one single approach, there is a trend to integrate both the outreach and inreach approaches to ensure that the screening services can be utilized by heterogeneous populations. The benefit of the integrated approach has been supported by our study, where an approximately 70% increase in the coverage rate was noted following the implementation of the inreach approach. Through the person-to-person counselling, such an approach may focus on the individual need of each subject and thus engage more subjects participating in the screening. Regarding the latter, efforts may include the analysis of sociocultural factors on adherence to screening and the use of reminders by telephone calls or specialized letters [42–44]. Although these interventions are undoubtedly important, they are unlikely to have the same impact as an intervention that provides services easily accessible to a population.

The integration of outreach and inreach approaches has been shown to be effective in promoting participation in cancer preventive services, such as screening for breast cancer, cervical cancer, CRC, and other cancers [18–20, 45–48]; however, outcome assessment has rarely been pursued. In the present study, we found that, in the Period 2, a higher proportion of men (an approximate 21% increase) and a higher hospital level in performing the confirmatory diagnostic examinations have been associated with a greater yield of CRCs. The increase in both early-stage and advanced-stage CRCs in Period 2 and the higher positivity rate of FIT, PPV for cancer detection, and cancer detection rate using the inreach approach support our original speculation that the inreach approach cannot only raise the inceptive of populations and improve their accessibility to screening but also include a higher proportion of subjects with clinical symptoms, which may be associated with advanced disease.

In the meanwhile, we found an approximately 270% increase in the number of positive tests but only an approximately 150% increase in the number of confirmatory diagnostic examinations, which has led to a decline in the referral rate such that a similar two-year sensitivity of the screening program (approximate 60%) was observed between the two periods. Furthermore, there was no change in the length of time between positive testing and confirmatory diagnostic examination. The finding was in contrast to our speculation that the referral rate in the inreach approach might be greater because of a non-fragmented referral process, which may be explained by the presence of extraneous factors, such as insufficient availability of colonoscopists in consideration of the rapid growth of the number of patients screened, an increase in the number of subjects with comorbidities, which sometimes made confirmatory diagnostic examination impractical, and erroneous use of FIT in subjects who had been screened already with other modalities.

Strengths of the present study include the large sample size, long follow-up time, execution on a nationwide scale, and registry of cancer incidence such that the outcome indicators could be thoroughly evaluated. In addition, our study evaluated the performance of a FIT-based CRC screening program, which is increasingly replacing guaiac-based tests on the global scale but is relatively deficient in a systematic evaluation of outcomes. However, our study has limitations. First, our study was based on the quasi-experimental design, where the results may be affected by the impact from the trend of increasing incidence of CRC as well as from the improved quality of screening tools (FIT) and diagnostic tools (colonoscopy). In addition, the response rate to invitation could not be accurately defined in our dataset, especially using the inreach approach. These factors are prone to bias the results in favor of the integrated approach. To overcome this limitation, we have evaluated which sub-populations may have responded to the new intervention by separating the posterior period according to the screening approaches of inreach and outreach; however, it is not possible to accurately separate subjects who were recruited from the outreach or inreach approach. Such a mixture may attenuate the pure impact from the inreach approach; however, we believe that the improvement related to the integrated approach did exist as the positivity rate of FIT and PPV for CRC detection were constantly seen in both univariate and multivariate analyses. Second, although the impact of decline in the referral rate appeared to be counterbalanced by an increase in the initial uptake rate, further investigation is needed to learn how to optimize the availability of colonoscopists and how to decrease the number of subjects who were unable to complete the screening process of FIT. Finally, the shorter follow-up time in the second period has made our analysis of CRC-specific mortality insufficient. Although the finding that a significant stage shifting of CRC may reasonably lead to a significant mortality reduction for both periods, further observation is needed to compare the difference in magnitude between these two approaches.

Through a systematic evaluation of outcome variables in the present study, the integration of inreach and outreach approaches has effectively decreased barriers for subjects in accessing to the screening service without affecting the main purpose of screening in the early detection of colonic neoplasms. Such integration is advisable in areas where a screening program is ongoing on the population scale.

## Supporting Information

S1 File Supporting information file 1. The S1 File contains the comparisons of different indicators between inreach and outreach screening approach during Peroid 2: Table A: Colonoscopy quality indicators stratified by the hospital levels in the nationwide colorectal cancer screening program. Table B: Baseline characteristics of the screened population during Period 2, stratified by the screening approaches. Table C: Numbers of tested population, positive tests, and confirmatory diagnoses stratified by the age and gender, and screening approaches during Period 2. Table D: Positive predictive values and detection rates for the advanced adenoma and colorectal cancer according to the age, gender, and screening approach during Period 2. Table E: Comparisons of the number of interval cancer, interval cancer rate, and test sensitivity between outreach and inreach approaches during Period 2. Table F: Comparisons of the test performance between two screening approaches during Period 2 using the Poisson regression models. Table G: Examples in the delivery methods for screening service in population-based CRC screening programs. Fig A: Incidence rates of CRC of the nationwide screening program based on biennial FIT, which are stratified by the screening approaches and the cancer detection modes during Period 2. Cancer was staged according to the American Joint Committee on Cancer (AJCC) 7th staging system.
(DOCX)

## Abbreviations

CRC: colorectal cancer
FIT: fecal immunochemical test
PPV: positive predictive value
AJCC: American Joint Committee on Cancer
ICD: International Classification of Diseases
RR: relative risk
CI: confidence interval
NHS: National Health Service
