# Supplementary material for: Outreach and Inreach Organized Service Screening Programs for Colorectal Cancer
Source: PLoS One. 2016 May 12;11(5):e0155276. doi: 10.1371/journal.pone.0155276 (PMC4865222; doi:10.1371/journal.pone.0155276)

**Supporting information file 1**

This supplementary data has been provided by the authors to give readers additional information about their work.

Supplement to: Chou CK, *et al*. Outreach and Inreach Organized Service Screening Programs for Colorectal Cancer

**Table A . Colonoscopy quality indicators stratified by the hospital levels in the nationwide colorectal cancer screening program**

| **Hospital level** | **Number** | **Colonoscopy as confirmatory diagnosis (%)** | **Cecal intubation rate (%)** | **Adenoma detection rate (%)** | **Advanced adenoma detection rate (%)** | **Resection rate of <2 cm adenoma (%)** |
| --- | --- | --- | --- | --- | --- | --- |
| Medical center | 42,399 | 88.6 | 91.3 | 52.1 | 16.9 | 94.8 |
| Regional hospital | 57,169 | 91.5 | 89.0 | 51.5 | 15.8 | 88.0 |
| Local hospital and clinic | 27,356 | 89.3 | 81.7 | 51.1 | 16.4 | 66.3 |

**Table B . Baseline characteristics of the screened population during Period 2, stratified by the screening approaches**

| **Characteristics** | **Outreach (*n*=960,103)** | **Inreach (*n*=1,242,898)** | ***P* value** |
| --- | --- | --- | --- |
| **Demographic characteristics** |  |  |  |
| Coverage rate (%) | 16.1 | 20.8 | <0.01 |
| Age, years (mean ± SD) | 57.44 ± 5.67 | 57.63 ± 5.60 | 0.98 |
| Gender, *n* (%) |  |  | <0.01 |
| Male | 441,248 (46.0) | 582,401 (46.9) |  |
| Female | 518,855 (54.0) | 660,497 (53.1) |  |
| **Geographic area, *n* (%)** |  |  | <0.01 |
| Northern area | 360,600 (37.6) | 578,157 (46.5) |  |
| Central area | 281,153 (29.3) | 233,687 (18.8) |  |
| Southern area | 243,755 (25.4) | 390,267 (31.4) |  |
| Eastern area and offshore island | 74,595 (7.7) | 40,787 (3.3) |  |
| **Fecal immunochemical test, *n* (%)** |  |  | <0.01 |
| OC-Sensor | 608,963 (63.4) | 773,401 (62.2) |  |
| HM-Jack | 351,138 (36.6) | 469,489 (37.8) |  |
| Others | 2 (0.0) | 8 (0.0) |  |
| **Confirmatory examination characteristics** |  |  |  |
| Time to confirmatory examination, months (mean ± SD) | 1.33 ± 1.61 | 1.29 ± 1.49 | 0.99 |
| Hospital level for confirmatory diagnosis, *n* (%) |  |  | <0.01 |
| Medical center | 12,376(30.4) | 21,237 (40.6) |  |
| Regional hospital | 19,968 (49.0) | 20,338 (38.9) |  |
| Local hospital and clinic | 8,127 (19.9) | 9,684 (18.5) |  |
| Non-specified | 295 (0.7) | 1,033 (2.0) |  |
| Confirmatory examination tool, *n* (%) |  |  | <0.01 |
| Colonoscopy | 37,624 (92.3) | 47,517 (90.9) |  |
| Sigmoidoscopy + barium enema | 3,020 (7.4) | 4,679 (8.9) |  |
| Missing data | 122 (0.3) | 96 (0.2) |  |
| Screened-detected cancer, *n* (per 1,000) | 3,146 (3.3) | 4,333 (3.5) | <0.01 |
| Colonoscopic quality indicator (%) |  |  |  |
| Cecal intubation rate* | 93.2 | 93.1 | 0.57 |
| Adenoma detection rate^†^ | 54.7 | 53.8 | <0.01 |
| Advanced adenoma detection rate^†^ | 16.8 | 17.4 | <0.05 |
| Resection rate of <2cm adenoma^‡^ | 90.0 | 92.2 | <0.01 |

*Cecal intubation rate was defined as the number of subjects with cecal intubation/the number of subjects screened with colonoscopy; ^†^(advanced) adenoma detection rate was defined as the number of subjects with at least one detected (advanced) adenoma /the number of subjects positive to FIT having attended a colonoscopy; ^‡^resection rate of <2cm adenoma was defined as the number of subjects with resection of adenoma/the number of subjects with at least one detected <2cm adenoma having attended a colonoscopy.

**Table C . Numbers of tested population, positive tests, and confirmatory diagnoses stratified by the age and gender, and screening approaches during Period 2**

|  | **Tested population** | | **Positive test** | | **Positivity rate (%)** | | **Diagnostic examination** | | **Referral rate for diagnostic examination (%)** | |
| --- | --- | --- | --- | --- | --- | --- | --- | --- | --- | --- |
|  | Outreach | Inreach | Outreach | Inreach | Outreach | Inreach | Outreach | Inreach | Outreach | Inreach |
| Male |  |  |  |  |  |  |  |  |  |  |
| 50–59 years | 287,512 | 375,763 | 24,467 | 33,509 | 8.5^†^ | 8.9^†^ | 13,798 | 17,555 | 56.4^†^ | 52.4^†^ |
| 60–69 years | 153,736 | 206,638 | 18,134 | 25,257 | 11.8^†^ | 12.2^†^ | 10,080 | 13,110 | 55.6^†^ | 51.9^†^ |
| Subtotal | 441,248 | 582,401 | 42,601 | 58,766 | 9.7^†^ | 10.1^†^ | 23,878 | 30,665 | 56.1^†^ | 52.2^†^ |
| Female |  |  |  |  |  |  |  |  |  |  |
| 50–59 years | 371,418 | 459,877 | 19,139 | 26,068 | 5.2^†^ | 5.7^†^ | 10,773 | 13,524 | 56.3^†^ | 51.9^†^ |
| 60–69 years | 147,437 | 200,620 | 11,459 | 16,597 | 7.8^†^ | 8.3^†^ | 6,115 | 8,103 | 53.4^†^ | 48.8^†^ |
| Subtotal | 518,855 | 660,497 | 30,598 | 42,665 | 5.9^†^ | 6.5^†^ | 16,888 | 21,627 | 55.2^†^ | 50.7^†^ |
| Both genders |  |  |  |  |  |  |  |  |  |  |
| 50–59 years | 658,930 | 835,640 | 43,606 | 59,577 | 6.6^†^ | 7.1^†^ | 24,571 | 31,079 | 56.3^†^ | 52.2^†^ |
| 60–69 years | 301,173 | 407,258 | 29,593 | 41,854 | 9.8^†^ | 10.3^†^ | 16,195 | 21,213 | 54.7^†^ | 50.7^†^ |
| Total | 960,103 | 1,242,898 | 73,199 | 101,431 | 7.6^†^ | 8.2^†^ | 40,766 | 52,292 | 55.7^†^ | 51.6^†^ |

**P*<0.05 or ^†^*P*<0.01 in the comparison between outreach and inreach periods.

**Table D . Positive predictive values and detection rates for the advanced adenoma and colorectal cancer according to the age, gender, and screening approach during Period 2**

|  | **Positive predictive value (%)** | | | | **Detection rate(per 1,000)** | | | |
| --- | --- | --- | --- | --- | --- | --- | --- | --- |
|  | Advanced adenoma | | Colorectal cancer | | Advanced adenoma | | Colorectal cancer | |
|  | Outreach | Inreach | Outreach | Inreach | Outreach | Inreach | Outreach | Inreach |
| Male |  |  |  |  |  |  |  |  |
| 50–59 years | 18.2 | 18.8 | 6.8 | 7.0 | 8.7 | 8.8 | 3.3 | 3.3 |
| 60–69 years | 19.8 | 20.4 | 10.3^†^ | 11.4^†^ | 13.0 | 12.9 | 6.7^†^ | 7.2^†^ |
| Subtotal | 18.9 | 19.5 | 8.3* | 8.9* | 10.2 | 10.2 | 4.5^†^ | 4.7^†^ |
| Female |  |  |  |  |  |  |  |  |
| 50–59 years | 10.2 | 10.5 | 6.1 | 6.6 | 3.0^†^ | 3.1^†^ | 1.8^†^ | 1.9^†^ |
| 60–69 years | 13.1 | 12.7 | 8.5 | 8.9 | 5.4^†^ | 5.1^†^ | 3.5 | 3.6 |
| Subtotal | 11.3 | 11.3 | 7.0 | 7.5 | 3.7 | 3.7 | 2.3^†^ | 2.4^†^ |
| Both genders |  |  |  |  |  |  |  |  |
| 50–59 years | 14.7 | 15.2 | 6.5 | 6.8 | 5.5^†^ | 5.6^†^ | 2.4^†^ | 2.5^†^ |
| 60–69 years | 17.3 | 17.4 | 9.6^†^ | 10.5^†^ | 9.3^†^ | 9.1^†^ | 5.2^†^ | 5.4^†^ |
| Total | 15.7 | 16.1 | 7.7^†^ | 8.3^†^ | 6.7^†^ | 6.8^†^ | 3.3^†^ | 3.5^†^ |

**P*<0.05 or ^†^*P*<0.01 in the comparison between outreach and inreach periods.

**Table E . Comparisons of the number of interval cancer, interval cancer rate, and test sensitivity between outreach and inreach approaches during Period 2**

|  | **Person-year at risk*** | | **No. of IC** | | **Incidence of IC**  **(expected incidence in the absence of screening)^†^** | | **Proportional incidence** | **Test sensitivity^1^, %**  **(95% CI)^‡^** | | **Two-year sensitivity^2^, % (95% CI)^‡^** | |
| --- | --- | --- | --- | --- | --- | --- | --- | --- | --- | --- | --- |
| Outreach |  | |  | |  | |  |  | |  | |
| Male | |  | |  |  |  | | |  | |  |
| 50–59 years | 509,171 | | 107 | | 21.0 (75.3) | | 0.28 | 72 (65-80) | | 57 (52-64) | |
| 60–69 years | 284,137 | | 120 | | 42.2 (180.3) | | 0.23 | 77 (70-83) | | 60 (55-65) | |
| Subtotal | 793,308 | | 227 | | 28.6 (112.9) | | 0.25 | 75 (70-80) | | 59 (55-63) | |
| Female |  | |  | |  | |  | | |  | |
| 50–59 years | 686,056 | | 105 | | 15.3 (55.0) | | 0.28 | 72 (65-80) | | 62 (56-68) | |
| 60–69 years | 280,070 | | 88 | | 31.4 (131.6) | | 0.24 | 76 (69-84) | | 66 (60-73) | |
| Subtotal | 966,126 | | 193 | | 20.0 (77.2) | | 0.26 | 74 (69-80) | | 64 (59-69) | |
| Both genders |  | |  | |  | |  |  | |  | |
| 50–59 years | 1,195,227 | | 212 | | 17.7 (65.0) | | 0.27 | 73 (68-78) | | 60 (56-65) | |
| 60–69 years | 564,207 | | 208 | | 36.9 (155.1) | | 0.24 | 76 (71-81) | | 62 (58-66) | |
| Total | 1,759,434 | | 420 | | 23.9 (93.9) | | 0.25 | 75 (71-78) | | 61 (58-64) | |
| Inreach |  | |  | |  | |  |  | |  | |
| Male |  | |  | |  | |  |  | |  | |
| 50–59 years | 642,974 | | 146 | | 22.7 (75.3) | | 0.30 | 70 (64-76) | | 51 (47-56) | |
| 60–69 years | 367,745 | | 153 | | 41.6 (180.3) | | 0.23 | 77 (71-83) | | 62 (58-67) | |
| Subtotal | 1,010,719 | | 299 | | 29.6 (113.5) | | 0.26 | 74 (70-78) | | 58 (55-61) | |
| Female |  | |  | |  | |  |  | |  | |
| 50–59 years | 815,755 | | 120 | | 14.7 (55.0) | | 0.27 | 73 (67-80) | | 60 (55-66) | |
| 60–69 years | 365,003 | | 91 | | 24.9 (131.6) | | 0.19 | 81 (74-89) | | 70 (64-76) | |
| Subtotal | 1,180,758 | | 211 | | 17.9 (78.7) | | 0.23 | 77 (72-82) | | 65 (61-69) | |
| Both genders |  | |  | |  | |  | | |  | |
| 50–59 years | 1,458,729 | | 266 | | 18.2 (65.0) | | 0.28 | 72 (68-77) | | 56 (53-60) | |
| 60–69 years | 732,748 | | 244 | | 33.3 (155.1) | | 0.21 | 79 (74-83) | | 65 (62-69) | |
| Total | 2,191,477 | | 510 | | 23.3 (95.1) | | 0.24 | 76 (72-79) | | 61 (59-64) | |

IC =interval cancer.

*The interval cancer was defined as a cancer that developed in the interval of 2 years following a negative FIT result. For those who had more than 2 years of follow-up but did not receive the subsequent screening, their follow-up time was set at 2 years in the calculation of person-years at risk.

^†^Per 100,000 person-years

^‡^Test sensitivity^1^ was generated from the number of interval cancer in the two-year period of observation following a negative FIT. Two-year sensitivity^2^ of the program was generated from the number of interval cancer in the two-year period of observation following a negative FIT or a positive FIT followed by a negative assessment or no further assessment.

**Table F . Comparisons of the test performance between two screening approaches during Period 2 using the Poisson regression models**

| **Model^*^** | **Relative risk** | **95% CI** |
| --- | --- | --- |
| Positive predictive value for advanced adenoma detection |  |  |
| Model 1 |  |  |
| Inreach *vs*. outreach | 1.02 | 0.99-1.06 |
| Model 2 |  |  |
| Inreach *vs*. outreach | 1.02 | 0.98-1.06 |
| Age 60–69 *vs.* 50–59 years | 1.13^†^ | 1.10-1.16 |
| Male *vs.* female | 1.68^†^ | 1.62-1.74 |
| OC-Sensor *vs.*HM-Jack | 1.22^†^ | 1.17-1.27 |
| Medical center/regional hospital *vs*. local hospital/clinic^‡^ | 0.93^†^ | 0.87-0.98 |
| Advanced adenoma detection rate |  |  |
| Model 1 |  |  |
| Inreach *vs*. outreach | 1.01 | 0.98-1.05 |
| Model 2 |  |  |
| Inreach *vs*. outreach | 0.98 | 0.91-1.05 |
| Age 60–69 *vs.* 50–59 years | 1.49^†^ | 1.44-1.54 |
| Male *vs.* female | 2.64^†^ | 2.53-2.76 |
| OC-Sensor *vs.*HM-Jack | 1.05 | 0.98-1.12 |
| Medical center/regional hospital *vs*. local hospital/clinic^‡^ | 3.94^†^ | 3.39-4.59 |
| Positive predictive value for cancer detection |  |  |
| Model 1 |  |  |
| Inreach *vs*. outreach | 1.07^†^ | 1.02-1.12 |
| Model 2 |  |  |
| Inreach *vs*. outreach | 1.07^†^ | 1.01-1.12 |
| Age 60–69 *vs.* 50–59 years | 1.48^†^ | 1.41-1.55 |
| Male *vs.* female | 1.17^†^ | 1.11-1.22 |
| OC-Sensor *vs.*HM-Jack | 1.27^†^ | 1.20-1.35 |
| Medical center/regional hospital *vs*. local hospital/clinic^‡^ | 1.12^†^ | 1.01-1.23 |
| Cancer detection rate |  |  |
| Model 1 |  |  |
| Inreach *vs*. outreach | 1.06^†^ | 1.02-1.11 |
| Model 2 |  |  |
| Inreach *vs*. outreach | 1.02 | 0.94-1.10 |
| Age 60–69 *vs.* 50–59 years | 2.01^†^ | 1.92-2.11 |
| Male *vs.* female | 1.76^†^ | 1.68-1.85 |
| OC-Sensor *vs.* HM-Jack | 1.06 | 0.98-1.16 |
| Medical center/regional hospital *vs*. local hospital/clinic^‡^ | 4.05^†^ | 3.56-4.62 |
| Interval cancer rate |  |  |
| Model 1 |  |  |
| Inreach *vs*. outreach | 0.97 | 0.86-1.11 |
| Model 2 |  |  |
| Inreach *vs*. outreach | 0.96 | 0.84-1.10 |
| Age 60–69 *vs.* 50–59 years | 1.88^†^ | 1.65-2.15 |
| Male *vs.* female | 1.49^†^ | 1.30-1.70 |
| OC-Sensor *vs.*HM-Jack | 0.98 | 0.86-1.12 |

^*^Model 1: the crude Poisson regression model; model 2: the multivariate Poisson regression model adjusted for the city/county clustering, age and gender distributions, brand of FIT, and the hospital levels^‡^ (a dichotomous predictor to represent the colonoscopy quality) for the positive predictive value and detection rate.

^†^*P*<0.05.

**Table G . Examples in the delivery methods for screening service in population-based CRC screening programs**

| **Area or country,**  **author, year** | **Primary screen method; eligible age** | **Delivery method** | **Screened number** | **Participation/ coverage (%)** | **Positivity rate (%)** | **Confirmatory diagnostic rate (%)** | **PPV for AA (%)** | **PPV for CRC (%)** | **AA detection rate (per 1000)** | **CRC detection rate (per 1000)** |
| --- | --- | --- | --- | --- | --- | --- | --- | --- | --- | --- |
| UK, Logan et al, 2012 [1] | Biennial 3 sets of gFOBT; aged 50–69 | Outreach approach:  Direct mailing with invitation letter and gFOBT; if no return: Reminder sent within 4 weeks. | 1.08 millions | 55–60/– | 0.6 | 83.0 | M:31.4  F:21.2 | M:11.6  F:7.8 | 6.1 | 1.6 |
| Selected areas, France  Leuraud et al, 2013 [2] | Biennial 2 sets of gFOBT; aged 50–74 | Outreach approach:  Mailing with invitation letter to visit their GP for screening; if no return: 1^st^ reminder, 2^nd^ reminder for positive tests, and 3^rd^ reminder for positive tests. | 3 millions | 34.3/– | 2.8 | 88.0 | M:24.4  F:14.2 | M:9.0  F:5.8 | 4.9 | 1.9 |
| Stockholm and Gotland, Sweden  Blom et al, 2014 [3] | Biennial 3 sets of gFOBT; aged 60–69 | Outreach approach:  Direct mailing with invitation letter and gFOBT with a prepaid return envelope; if no return, reminder sent within 8 weeks. | 206,056 | 57–64/– | 2.1 | 88.3 | Adenoma, 21.7–38.7 | 5.6 | – | 1.0 |
| Finland, Malila et al, 2011 [4] | Biennial 3 sets of gFOBT; aged 60–69 | Outreach approach:  Direct mailing with invitation letter and gFOBT. | 74,592 | –/68 | 2.4–2.9 | 90 | Adenoma, 33.2-38.2 | 7.5–4.3 | – | 0.9–2.1 |
| Selected areas, Spain Ricardo-Rodrigues et al, 2014 [5], Miia et al, 2012 [6] and Ascunce et al, 2010 [7] | Biennial gFOBT or FIT; aged 50–69 | Outreach approach:  Mailing with invitation letter with information how to obtain FOBT test. | 418,973 [7] | 17.2–59.0 [7]/7.7 [8] | 1.7–9.5 [7] | 89.8^†^ | 21.2^*^ | 6.2* | 7.2* | 1.7–3.4 [4] |
| Regional programs, Italy  Zorzi et al, 2014 [9, 10] | Biennial FIT; aged 50–69 | Outreach and inreach approaches:  Mailing with invitation letter and invitation from GP when clinic visit. | 3.4 millions | 48/29 [11] | 5.5 | 81.4 | – | – | 10.3 | 2.4 |
| Canada Major et al, 2013 [12, 13] | Biennial FIT or gFOBT; aged 50–74 | Outreach and inreach approaches:  Initiated by media trigger and doctor visit, invitation.  Inreach: stool kit available at PCP.  Outreach: stool kit from a pharmacist or by telephone calling (Ontario). | 104,750 | –/16.1 | 4.4 | 80.5 | Adenoma, gFOBT:35.9; FIT: 50.6 | – | Adenoma,16.8 | 1.8 |
| USA, Colorectal Cancer Control Program (25 states and 4 tribes)  Joseph et al. 2011 and CDC, 2012 and 2014 [14, 15, 16] | High-sensitivity g-FOBT, FIT, sigmoidoscopy, or colonoscopy; aged 50–75 | Outreach and inreach approaches:  Providing CRC screening services to uninsured and underinsured low-income adults.  Promoting population-wide CRC screening through evidence-based interventions identified in the Guide to Community Preventive Services. | Survey | 65.1/– | – | – | – | – | – | – |
| USA, Kaiser Permanente  Moiel et al. 2011 [17] | High-sensitivity g-FOBT, FIT, sigmoidoscopy, or colonoscopy; aged 50–75 | Outreach and inreach approaches:  Directing mailing FIT Kits to members.  Providing screening service during office visit. | No. of members: 3.6  millions | –/49.5 | – | – | – | – | – | – |
| Korea Shim et al, 2010 [18]  Choi et al, 2012 [19] | annual FIT;  aged 50 and more | Outreach approach:  Mailing with invitation letter | 984,915 | 21.2/– | 7.5 | 61.3 | – | – | – | – |
| Taiwan  Chiang et al,  2014 [20]  Chiu et al,  2015 [21] | National program; biennial FIT;  aged 50–69  (50–74 from June, 2013) | Outreach and inreach approaches:  Period 1 (2004–2009): outreach approach by local health unit with invitation by phone, health promotion activities.  Period 2 (2010–now): combined with the hospital/clinic-based in-reach approach. | 3.4 millions | –/36.3 | 7.9 | 53.3 | 15.9 | 8.0 | 6.7 | 3.4 |

Abbreviation: CRC, colorectal cancer; AA, advanced adenoma; gFOBT, guaiac-based fecal occult blood test; PCP, primary care provider; PPV, positive predictive value; FIT, fecal immunochemical test; GP, general practitioner.

^*^Data from Catalonia, where the screening was based on gFOBT.

**Reference for Table G**

1. Logan RF, Patnick J, Nickerson C, Coleman L, Rutter MD, von Wagner C, et al. Outcomes of the Bowel Cancer Screening Programme (BCSP) in England after the first 1 million tests. Gut. 2012;61(10):1439-46. doi: 10.1136/gutjnl-2011-300843. PubMed PMID: 22156981; PubMed Central PMCID: PMC3437782.
2. Leuraud K, Jezewski-Serra D, Viguier J, Salines E. Colorectal cancer screening by guaiac faecal occult blood test in France: Evaluation of the programme two years after launching. Cancer epidemiology. 2013;37(6):959-67. doi: 10.1016/j.canep.2013.07.008. PubMed PMID: 24035240.
3. Blom J, Kilpelainen S, Hultcrantz R, Tornberg S. Five-year experience of organized colorectal cancer screening in a Swedish population - increased compliance with age, female gender, and subsequent screening round. Journal of medical screening. 2014;21(3):144-50. doi: 10.1177/0969141314545555. PubMed PMID: 25070434.
4. Malila N, Palva T, Malminiemi O, Paimela H, Anttila A, Hakulinen T, et al. Coverage and performance of colorectal cancer screening with the faecal occult blood test in Finland. Journal of medical screening. 2011;18(1):18-23. doi: 10.1258/jms.2010.010036. PubMed PMID: 21536812.
5. Ricardo-Rodrigues I, Jimenez-Garcia R, Hernandez-Barrera V, Carrasco-Garrido P, Jimenez-Trujillo I, Lopez-de-Andres A. Adherence to and predictors of participation in colorectal cancer screening with faecal occult blood testing in Spain, 2009-2011. European journal of cancer prevention : the official journal of the European Cancer Prevention Organisation. 2014. doi: 10.1097/CEJ.0000000000000088. PubMed PMID: 25304027.
6. Mila N, Garcia M, Binefa G, Borras JM, Espinas JA, Moreno V. [Adherence to a population-based colorectal cancer screening program in Catalonia (Spain), 2000-2008]. Gaceta sanitaria. 2012;26(3):217-22. doi: 10.1016/j.gaceta.2011.10.020. PubMed PMID: 22361637.
7. Ascunce N, Salas D, Zubizarreta R, Almazan R, Ibanez J, Ederra M, et al. Cancer screening in Spain. Annals of oncology : official journal of the European Society for Medical Oncology / ESMO. 2010;21 Suppl 3:iii43-51. doi: 10.1093/annonc/mdq085. PubMed PMID: 20427360.
8. Shelton RC, Jandorf L, Ellison J, Villagra C, DuHamel KN. The influence of sociocultural factors on colonoscopy and FOBT screening adherence among low-income Hispanics. Journal of health care for the poor and underserved. 2011;22(3):925-44. doi: 10.1353/hpu.2011.0074. PubMed PMID: 21841288; PubMed Central PMCID: PMC3635094.
9. Zorzi M, Fedeli U, Schievano E, Bovo E, Guzzinati S, Baracco S, et al. Impact on colorectal cancer mortality of screening programmes based on the faecal immunochemical test. Gut. 2014. doi: 10.1136/gutjnl-2014-307508. PubMed PMID: 25179811.
10. Zorzi M, Fedato C, Grazzini G, Sassoli de' Bianchi P, Naldoni C, Pendenza M, et al. [Screening for colorectal cancer in Italy, 2010 survey]. Epidemiologia e prevenzione. 2012;36(6 Suppl 1):55-77. PubMed PMID: 23293271.
11. Moss SM, Campbell C, Melia J, Coleman D, Smith S, Parker R, et al. Performance measures in three rounds of the English bowel cancer screening pilot. Gut. 2012;61(1):101-7. doi: 10.1136/gut.2010.236430. PubMed PMID: 21561880; PubMed Central PMCID: PMC3230830.
12. Major D, Bryant H, Delaney M, Fekete S, Gentile L, Harrison M, et al. Colorectal cancer screening in Canada: results from the first round of screening for five provincial programs. Current oncology. 2013;20(5):252-7. doi: 10.3747/co.20.1646. PubMed PMID: 24155629; PubMed Central PMCID: PMC3805400.
13. ColonCancerCheck,Ontario,Canada.Available at: http://health.gov.on.ca/en/public/programs/coloncancercheck/ Accessed Febuary 8, 2015.
14. Centers for Disease C, Prevention. Vital signs: colorectal cancer screening test use--United States, 2012. MMWR Morbidity and mortality weekly report. 2013;62(44):881-8. PubMed PMID: 24196665.
15. Joseph DA, DeGroff AS, Hayes NS, Wong FL, Plescia M. The Colorectal Cancer Control Program: partnering to increase population level screening. Gastrointestinal endoscopy. 2011;73(3):429-34. doi: 10.1016/j.gie.2010.12.027. PubMed PMID: 21353839.
16. Colorectal Cancer Control Program. Available at: http://www.cdc.gov/cancer/crccp/ Accessed Febuary 8, 2015
17. Moiel D, Thompson J. Early detection of colon cancer-the kaiser permanente northwest 30-year history: how do we measure success? Is it the test, the number of tests, the stage, or the percentage of screen-detected patients? The Permanente journal. 2011;15(4):30-8. PubMed PMID: 22319413; PubMed Central PMCID: PMC3267557.
18. Shim JI, Kim Y, Han MA, Lee HY, Choi KS, Jun JK, et al. Results of colorectal cancer screening of the national cancer screening program in Korea, 2008. Cancer research and treatment : official journal of Korean Cancer Association. 2010;42(4):191-8. doi: 10.4143/crt.2010.42.4.191. PubMed PMID: 21253320; PubMed Central PMCID: PMC3021737
19. Choi KS, Lee HY, Jun JK, Shin A, Park EC. Adherence to follow-up after a positive fecal occult blood test in an organized colorectal cancer screening program in Korea, 2004-2008. Journal of gastroenterology and hepatology. 2012;27(6):1070-7. doi: 10.1111/j.1440-1746.2011.06944.x. PubMed PMID: 22004224.
20. Chiang TH, Chuang SL, Chen SL, Chiu HM, Yen AM, Chiu SY, et al. Difference in performance of fecal immunochemical tests with the same hemoglobin cutoff concentration in a nationwide colorectal cancer screening program. Gastroenterology. 2014;147(6):1317-26. doi: 10.1053/j.gastro.2014.08.043. PubMed PMID: 25200099.
21. Chiu HM, Chen SL, Yen AM, Chiu SY, Fann JC, Lee YC, et al. Effectiveness of fecal immunochemical testing in reducing colorectal cancer mortality from the One Million Taiwanese Screening Program. Cancer. 2015;121(18):3221-9. doi: 10.1002/cncr.29462. PubMed PMID: 25995082.

**Figure A .** Incidence rates of CRC of the nationwide screening program based on biennial FIT, which are stratified by the screening approaches and the cancer detection modes during Period 2. Cancer was staged according to the American Joint Committee on Cancer (AJCC) 7^th^ staging system.


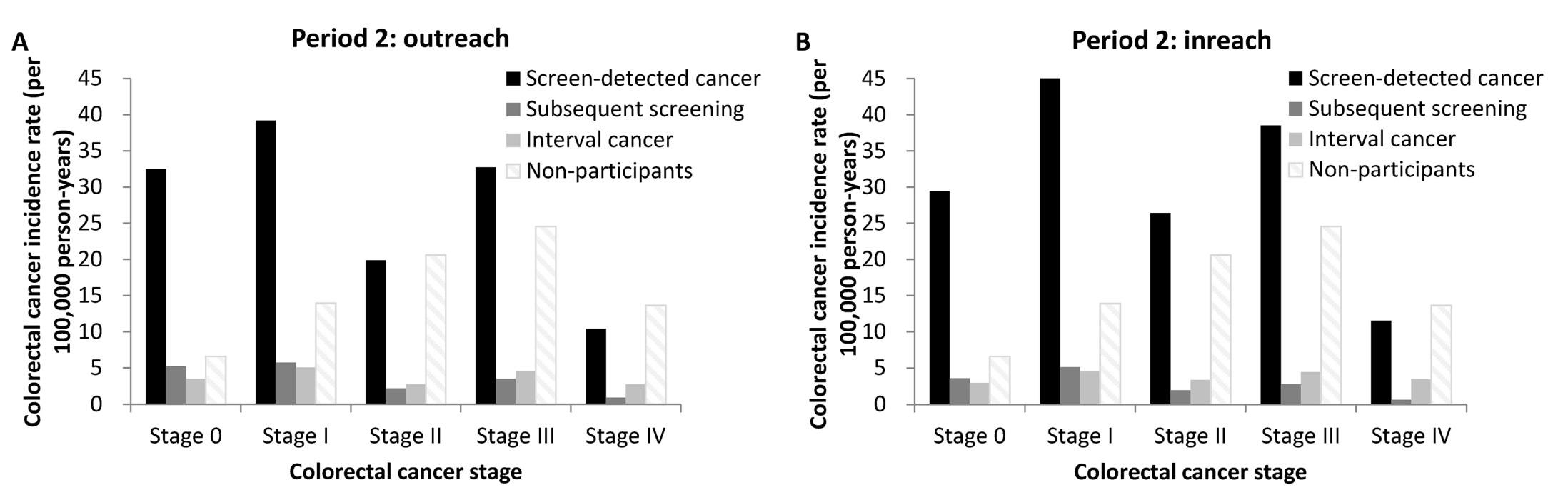

Supplement: S1 File — The S1 File contains the comparisons of different indicators between inreach and outreach screening approach during Peroid 2: Table A: Colonoscopy quality indicators stratified by the hospital levels in the nationwide colorectal cancer screening program. Table B: Baseline characteristics of the screened population during Period 2, stratified by the screening approaches. Table C: Numbers of tested population, positive tests, and confirmatory diagnoses stratified by the age and gender, and screening approaches during Period 2. Table D: Positive predictive values and detection rates for the advanced adenoma and colorectal cancer according to the age, gender, and screening approach during Period 2. Table E: Comparisons of the number of interval cancer, interval cancer rate, and test sensitivity between outreach and inreach approaches during Period 2. Table F: Comparisons of the test performance between two screening approaches during Period 2 using the Poisson regression models. Table G: Examples in the delivery methods for screening service in population-based CRC screening programs. Fig A: Incidence rates of CRC of the nationwide screening program based on biennial FIT, which are stratified by the screening approaches and the cancer detection modes during Period 2. Cancer was staged according to the American Joint Committee on Cancer (AJCC) 7th staging system. (DOCX) [file pone.0155276.s001.docx]
